# Electrograms peak frequency analysis for ventricular tachycardia ablations: when technology improves our understanding of the physiology

**DOI:** 10.1093/europace/euae286

**Published:** 2024-11-11

**Authors:** Nicolò Martini, Federico Calore, Filippo Maria Cauti

**Affiliations:** Department of Cardiac, Thoracic and Vascular Sciences and Public Health, University of Padova, Padova, Italy; Cardiology Unit, S. Maria del Prato Hospital, Feltre, Italy; Abbott Medical Italy, Sesto San Giovanni, Milan, Italy; Arrhythmology Department, IRCCS San Raffaele Hospital, Milan, Italy

We have read the paper by Tonko *et al*.^1^ with great interest. In this retrospective study, the authors compared the near field (NF) detection algorithm to first deflection and last deflection-based maps of 18 patients affected by scar-related ventricular tachycardia (VT). Thanks to the NF algorithm, they could characterize the three-dimensional nature of VT circuits with a better identification of intramural components. Nevertheless, peak frequency (PF) analysis did not allow the discrimination of functionally critical sites for VT maintenance from bystander sites. The present study further highlights what lies behind re-entrant VT circuits, which are generally overly simplified in a bi-dimensional representation by current mapping systems. Ventricular tachycardia circuits are three-dimensional, and if VT activation mapping and diastolic pathway recordings still play a pivotal role in VT ablation, the functional analysis and characterization of the ventricular electrical activity are crucial in improving the intra-procedural and long-term outcomes.^2,3^ Despite its clinical usefulness, PF analysis provides a more in-depth understanding of the electrical impulse pathway, especially inside the isthmus, enhancing vulnerable areas inside the circuit.^2,4,5^ As Cauti *et al*.,^6^ we have recently provided a thorough analysis of electrogram (EGM) PF during VT activation mapping in a large series. Our article further stressed the importance of the evaluation of the VT EGM frequency spectrum, supplying accurate data about the ‘3D nonlinear’ wavefront propagation of the VT electrical activity among the different layers of the ventricular myocardium, both in depth and width. We have demonstrated that EGMs with the highest frequency belong to more superficial, surviving myocytes within a VT isthmus and can be used as a more accessible and vulnerable spot within the isthmus itself. As a proof of concept, radiofrequency (RF) delivery at the highest frequencies inside slow conductive channels allowed an instantaneous VT termination regardless of the diastolic phase.^6^ Differently, low-frequency EGMs represented intramural/epicardial components, and longer RF applications were necessary to get a delayed VT interruption. As already demonstrated by Tung *et al*. and Nishimura *et al*.,^7,8^ our study confirmed that the diastolic pathway of a VT circuit might no longer be described solely as entry, mid-isthmus, and exit, as the electrical impulse can also cross the entire myocardial wall from the mapping surface to the opposite side. We have provided a simple but accurate way to deeply analyse and characterize the EGM spectrum within a VT activation mapping, going beyond the bi-dimensional representation of a VT circuit in such a way as to rapidly identify those areas that are critical for VT maintenance and whose RF target allows a fast VT interruption that may be mandatory in those patients with haemodynamic instability. As in our paper and the study of Natale *et al*.,^9^ analysis and characterization of the EGM spectrum represent the key to deeply understand VT behaviour and improve the intra-procedural and long-term outcomes of patients with scar-related VT. These findings and concepts perfectly align with the progress and the prospect of important improvement in VT ablation in recent decades.
